# Solution scattering at the Life Science X-ray Scattering (LiX) beamline

**DOI:** 10.1107/S1600577520002362

**Published:** 2020-03-31

**Authors:** Lin Yang, Stephen Antonelli, Shirish Chodankar, James Byrnes, Edwin Lazo, Kun Qian

**Affiliations:** a NSLS-II, Brookhaven National Laboratory, Upton, NY 11973, USA

**Keywords:** SAXS, WAXS, solution scattering, SEC-SAXS, automation

## Abstract

This work reports the instrumentation and software implementation at the Life Science X-ray Scattering (LiX) beamline at NSLS-II in support of biomolecular solution scattering. For automated static measurements, samples are stored in PCR tubes and grouped in 18-position sample holders. Unattended operations are enabled using a six-axis robot that exchanges sample holders between a storage box and a sample handler, transporting samples from the PCR tubes to the X-ray beam for scattering measurements. The storage box has a capacity of 20 sample holders. At full capacity, the measurements on all samples last for ∼9 h. For in-line size-exclusion chromatography, the beamline-control software coordinates with a commercial high-performance liquid chromatography (HPLC) system to measure multiple samples in batch mode. The beamline can switch between static and HPLC measurements instantaneously. In all measurements, the scattering data span a wide *q*-range of typically 0.006–3.2 Å^−1^. Functionalities in the Python package *py4xs* have been developed to support automated data processing, including azimuthal averaging, merging data from multiple detectors, buffer scattering subtraction, data storage in HDF5 format and exporting the final data in a three-column text format that is acceptable by most data analysis tools. These functionalities have been integrated into graphical user interfaces that run in *Jupyter* notebooks, with hooks for external data analysis software.

## Abstract 2

Instrumentation and data processing for solution scattering at the LiX beamline are presented.

## 1. Introduction   

The Life Science X-ray Scattering (LiX) beamline was constructed at NSLS-II as part of the Advanced Beamlines for Biological Investigations using X-rays (ABBIX) project (Fuchs *et al.*, 2014 ▸; DiFabio *et al.*, 2016 ▸) funded by the National Institute of Health. After beamline construction was completed at the end of 2015, technical commissioning and performance optimization of optical components followed (Chodankar & Yang, in preparation ▸). The beamline started supporting user experiments in August 2016, as the development of the experimental station continued. At present, 80% of the total beam time at the beamline is devoted to user experiments, mainly in the areas of solution scattering and microbeam structural mapping. User access is currently available in several modes (see https://www.bnl.gov/ps for details), ranging from the one-time-only same-cycle rapid access to the block allocation groups (BAGs) which offer two-year continuous access for groups of experimenters with coherent research interests. We also have a joint access program with the bioSANS beamline at the High Flux Isotope Reactor of Oakridge National Laboratory, from which users can obtain neutron and X-ray beam time under a single proposal.

From the outset, the LiX beamline was designed to support research in multiple scientific areas. A two-stage optical scheme was therefore adopted to enable rapid beam focus changes (DiFabio *et al.*, 2016 ▸). Briefly, the first stage of the optical system delivers a focused, monochromatic X-ray beam to the secondary source aperture (SSA), which is then imaged to the sample position using compound refractive lenses (CRLs) installed in a transfocator. The CRLs allow for instantaneous adjustment of the beam size at the sample position, based on experiment-specific requirements. The beam size and divergence are controlled by a combination of the SSA size, CRL configuration in the transfocator, location of the transfocator (variable distance from the sample) and the size of the divergence-defining aperture (DDA; located just upstream of the transfocator). In solution scattering measurements, the typical beam size is ∼0.5 mm × 0.5 mm, with the intention of spreading out the radiation dose from the X-ray beam into a large volume to mitigate radiation damage.

The experimental instrumentation consists of multiple experimental modules that are each designed for a specific type of measurement, but share the same mechanical interface to a common support to enable rapid module exchange with good positional repeatability. Initially we planned modules for in-vacuum tender X-ray measurements and reflectivity measurements, with kinematic mounts to interface with the large granite bench in the experimental station. As we focus the scope of the study to solution scattering and microbeam mapping, which are both conducted in transmission geometry with the samples under ambient pressure and requiring similar coarse positioning, the common interface for these experimental modules is now provided by a coarse positioning stage with *x* (horizontal and perpendicular to the beam) and *z* (along the beam) degrees of freedom. Dowel pins on the mounting interface are used to ensure positional repeatability during module exchange.

Scattering data are collected simultaneously on three Pilatus3 pixel array detectors, with overlaps between the ranges of scattering angles covered by the individual detectors. Combined, the scattering data typically span a continuous range of scattering vectors, *q* = 4πsinθ/λ, from 0.006 Å^−1^ to 3.2 Å^−1^ (see examples in Section 4), sufficient for practically all types of solution samples. Within this *q*-range, the water scattering peak centered at ∼2.0 Å^−1^ is fully visible, providing an internal reference for precise subtraction of buffer scattering, without requiring knowledge of the protein concentration in the solution (Yang, 2013 ▸).

Here we describe the instrumentation for automated static measurements and in-line size-exclusion chromatography (SEC) as well as the software for data collection and processing.

## 2. Instrumentation design   

There were several objectives when we designed the solution scattering instrumentation. Since the beamline supports multiple types of experiments, it is of great importance that the experimental module for solution scattering can be set up and removed rapidly. For this reason, we opted to decouple the sample cell (described below) from the vacuum path, instead of using the sample cell windows as vacuum windows, as is typically done at solution scattering beamlines (*e.g.* at beamline X9 of NSLS; Allaire & Yang, 2011 ▸). This increases the number of windows in the X-ray path from two to four. X-rays must also pass through air gaps with a total path length of ∼10 mm. In practice, we found that the scattering background was reasonable and did not degrade the quality of the data.

In order to cover a wide *q*-range in the scattering data using our in-vacuum wide-angle X-ray scattering (WAXS) detectors, we must position the sample cell very close to the vacuum window leading to the detector chamber. This constrains our options in locating the liquid handling equipment for transporting the samples to the flow cell, and certainly rules out utilizing designs (*e.g.* Round *et al.*, 2015 ▸) in which the samples are stored in close proximity to the sample cell to minimize sample transport time.

Finally, for in-line chromatography measurements it is important to preserve the resolution from the purification system. Therefore, the distance must be minimized between the sample cell, where the scattering measurements take place, and the high-performance liquid chromatography (HPLC) system.

**Figure 1:**
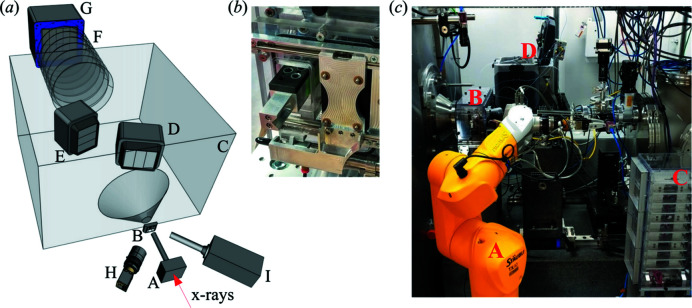
(*a*) Overall layout of the solution scattering instrumentation at LiX, with the following components: (A) guard slits, (B) flow cell, (C) WAXS chamber, (D) and (E) WAXS detectors, (F) bellow-based SAXS flight path, (G) SAXS detector, and (I) fluorescence detector. A mica-window-terminated beam tube extends the vacuum from the guard slits to just upstream of the flow cell. Another mica window on the nose cone on the WAXS chamber terminates the vacuum in the detector chamber. (*b*) Photograph of the PCR tube holder being partially loaded into the solution sample handler. Two PCR tubes are visible. (*c*) Photograph of the complete solution scattering setup at LiX: (A) Staubli six-axis robot, (B) solution scattering sample handler, (C) sample storage unit and (D) HPLC system. The X-ray beam enters from the right. As a reference for scale, the sample storage unit is 45 cm high, 18 cm wide (along the beam direction) and 14 cm deep.

Given these considerations, we have designed a system (Fig. 1 ▸) that consists of a sample handler for static measurements with its own controller, and a commercial Shimadzu HPLC system. The sample handler contains a three-channel flow cell to support both modes of measurements, as described below.

### 2.1. Flow cell   

All solution-scattering measurements are conducted using a flow-through cell to mitigate radiation damage to the sample. The cell consists of three flow channels: two of them are used for static measurements, matching the double-row format of the PCR tube holder as described below; the third channel is dedicated to in-line liquid chromatography (LC). Switching between the two modes is instantaneous by simply moving the required flow channel into the beam. Typically the flow cells used for solution scattering are based on glass capillaries and must be kept in a fixed position to avoid variations in the X-ray path length through the sample. This is not a significant concern in our case, owing to a flow cell design that features parallel flat mica windows and therefore ensures a constant X-ray path length, any variation of which can be accounted for using the buffer subtraction method based on the water scattering peak intensity described in Section 4.

**Figure 2:**
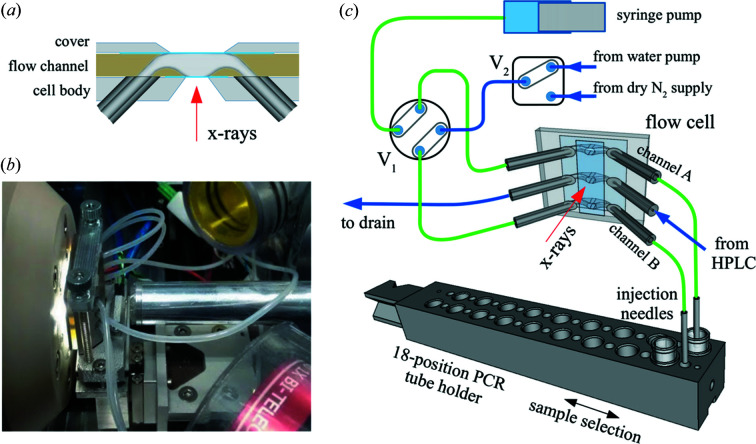
(*a*) Schematic showing the assembly of the flow cell from its components. The mica windows are shown as blue lines. The thickness of the flow channel (X-ray path length) is 1.5 mm. (*b*) Photograph of the flow cell installed at the beamline. The flow channels can be seen illuminated by LEDs. Also visible are the vacuum beam path (aluminium tube in the middle), the WAXS nose cone (beige, on the left) and the camera lens (red). (*c*) Details of the fluid connections in the sample handler. For clarity, only two PCR tubes are shown. The blue flow paths are one-way only, while fluids in the green paths can flow in both directions. Two valves are used. V_1_ is a four-port, two-position valve. In the position shown, the syringe pump has access to one of the PCR tubes through the top channel in the flow cell. The syringe pump and bottom channel are ready to be washed and dried, depending on the position of the two-position selection valve V_2_. The center-to-center distance between neighboring channels is 4.5 mm. The smaller holes between the PCR tubes in each row are the blank holes described in the text; one is under the upstream injection needle connected to channel B in the figure.

The flow cell is assembled [Fig. 2(*a*) ▸] from several components held together by screws: the cell body to which flow tubes are attached, the upstream mica window, the flow channels, the downstream mica window and the cover. Both the cover and the cell body are made of stainless steel to provide rigidity and chemical resistance. The mica windows (∼20 µm thick) enclose the flow channels to form the flow paths, which are then connected to the flow tubes. These tubes are glued into the cell body using a UV glue (Loctite AA3922), cured using a UV lamp after it has uniformly spread between the tubes and the cell body. The protruding ends of the tubes are then cut flush to the cell body surface. We experimented with several approaches of fabricating the flow channels. Our current approach is to 3D print the flow channels using a Formlabs Form 2 printer and its Durable Resin. The final print is uncured and therefore remains mildly flexible. The surface is polished to ensure good seals between the flow channels, the mica windows and the cell body. The thickness of the flow channel is 1.5 mm and defines the X-ray path length through the sample.

We found it critical to maintain a smooth flow path for the sample. Bubbles were often observed in earlier iterations of the flow cell. We eventually traced the issue to the mis­alignment of the flow channels and the flow tubes due to fabrication errors, which in turn result in step changes in the cross-section along the flow path. The flow channel in use is monitored constantly via a video camera. We use the live image to trigger data collection during static measurements, as discussed below in the data collection section (Section 3). This mechanism could also be used to flag scattering patterns for which bubbles were observed during data collection.

Temperature control for the sample cell is implemented using a Peltier cooler installed under the stainless cell body, with a water-cooled block as the heat sink. The sample cell can reach a nominal temperature range of 0–80°C. However, since the samples are not expected to reside in the sample cell for very long, the temperature of the environment around the sample cell also matters. We therefore also implemented cooling in the sample handler using circulating coolant from a chiller and in the sample storage unit for static measurements using Peltier coolers. We recommend our users to limit the sample temperature in the range 5–50°C. For static measurements, the sample can be optionally incubated in the sample cell for a user-defined period before the measurement.

### 2.2. High-throughput static measurements   

For static measurements, samples are stored in PCR tubes and grouped into 18-position sample holders. Each sample is transported from the PCR tube to the sample cell for measurement, and then returned to the original PCR tube upon completion. The sample cell is then washed with water at a high flow rate (∼50 µl s^−1^) and dried using nitro­gen gas. The water and nitro­gen are captured in a drain well located on the support for the PCR tube holder, and subsequently purged into a waste container by the negative pressure created by a vacuum pump. This process is accomplished using a syringe pump (Kloehn V6), a diaphragm water pump (KNF NF10) and a series of solenoid valves, schematically shown in Fig. 2(*c*) ▸. We intentionally keep the sample injection needles stationary and the PCR tube holder moveable to avoid potential sample loading issues in the event that the fluid connections between the sample cell and injection needles become faulty under repetitive motion.

Transporting samples between the PCR tube and the flow channel is time-consuming, as is washing and drying the flow channels to be ready for the next measurement. A typical measurement lasts ∼90 s, of which only a few seconds are spent on the actual scattering data collection. To speed up measurements, we arrange the samples in two rows and use two injection needles, so that the processes of washing/drying and transporting/measuring can take place in different flow channels at the same time. The measurement time per sample is ∼40% shorter using this concurrent method, compared with the sequential method in which cleaning and sample measurement occur in a single-threaded process. The two rows of PCR tubes are staggered. Therefore, when the sample holder is elevated by a pneumatic actuator to immerse one of the injection needles into the sample, the other needle goes into a blank hole in the sample holder and is hence uncontaminated. To avoid collision between the needles with the sample holder or PCR tubes, we use an optical sensor and a marker strip (black markers on a transparent substrate) to ensure that the needle and PCR tube are aligned before the pneumatic actuator is allowed to move the sample holder up.

To support fully automated measurements, we have installed a Staubli TX40 six-axis robot and a sample storage unit with a capacity of 20 sample holders [Fig. 1(*c*) ▸]. The TX40 has been programmed to exchange sample holders between the solution scattering sample handler and the storage unit, enabling continuous measurements on up to 360 samples (a total of ∼9 h using the concurrent method described above). We chose to use the Staubli robot to take advantage of existing software and expertise (*e.g.* use of an integrated force-torque sensor to detect collisions during operation) from our protein crystallography colleagues. On the other hand, we have incorporated design features to increase the tolerance for small positioning errors. The robot gripper is ‘self-centering’, with a half cylinder mounted on one finger and a half sphere on the other, interfacing with the Vee and cone features at the end of the PCR tube holder. Furthermore, the PCR tube holder has a guide groove on the bottom. The pins inside the sample storage unit and the sample handler therefore help guide the PCR tube holder to allow for more ‘sloppy’ positioning. There are also sensors that indicate the presence of a PCR tube holder at each possible location, to prevent the robot from picking up from an empty location or delivering to a location that is already occupied.

### 2.3. In-line size-exclusion chromatography   

The HPLC system is equipped with its own auto-sampler. It makes use of two high-pressure pumps (LC-10Ai) and a ten-port valve to work with two columns, such that while one column is in use the second column can be regenerated. In order to accommodate experiments that require the samples to be kept below ambient temperature, we installed the columns in a cooler together with the buffer reservoirs. The cooler can be filled with ice and stay cold for days. Cold blocks can also be installed in the HPLC auto sampler to keep the samples cold before the measurements.

Two detectors are installed in the HPLC system, for ultraviolet (UV, model SPD-20AV) absorbance and refractive index (RI, model RID-20 A) measurements. The eluent from the column is split into two flows (following Graewert *et al.*, 2015 ▸), one to the X-ray scattering flow cell and the other to the UV and RI detectors, which are connected in series. The splitting ratio between the two flows is ∼3:1. This is verified periodically by comparing the amounts of liquids collected at the exit of the waste lines. We are implementing real time monitoring using optical sensors that measure the time interval between the waste droplets.

The chromatographic resolution is an important consideration for in-line SEC measurements. The proteins in the eluent diffuse while being transported to the point of detection, resulting in broadening of the peaks in the chromatograms. We therefore locate the LC system close to the flow cell for the X-ray scattering measurements. The length of the PEEK tubing (0.01 inch inner diameter) between the HPLC system and the flow cell is <1 m, corresponding to an internal volume of <50 µl (minimal compared with the bed volume of the column and comparable to the sample load volume) and transit time of <6 s at a flow rate of 0.5 ml min^−1^.

**Figure 3:**
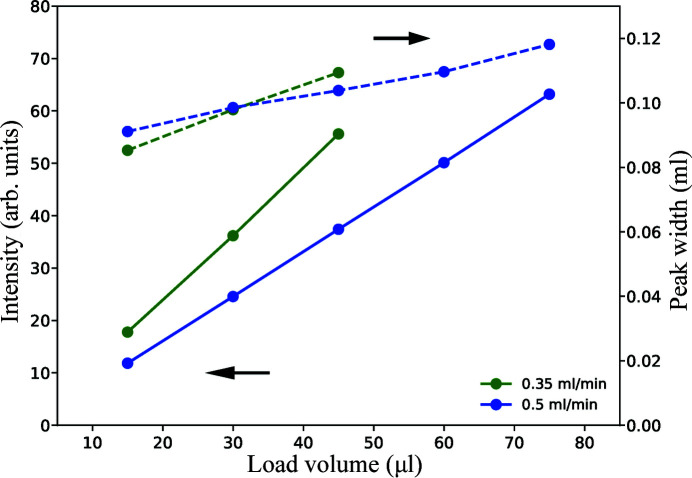
Characterization of the contribution to SEC chromatographic resolution due to the flow cell for X-ray scattering measurements. The data are obtained by flowing the sample directly to the sample cell with the column bypassed using a union. The peaks width (dashed lines) in the chromatogram increases, or the resolution degrades, as the sample load volume is increased. However, the corresponding increase in the scattering intensity (solid lines) outpaces the resolution degradation. This provides a basis for selecting an optimal sample load volume, which should maximize the scattering intensity without compromising the separation required in the chromatogram.

The cross-section of the flow channel in the sample cell (∼1 mm^2^) is considerably larger than that of the PEEK tubing used for HPLC (0.25 mm diameter). The flow cell itself is therefore another potential source of peak broadening. We characterized the ‘intrinsic resolution’ of the flow cell by flowing the sample directly to the sample cell, bypassing the column. We found the peak width to be similar to those in the chromatograms obtained when running the HPLC system offline (all the samples going to the UV/RI detectors) with the same sample load volume and flow rate, which is expected given the comparable internal volume of the flow cells (∼10 µl for X-ray, 12 µl for UV and 9 µl for RI, according to the equipment manuals). As the sample load volume is increased (Fig. 3 ▸), the peak intensity detected by X-ray scattering increases and the peak in the chromatogram becomes broader. However, chromatographic resolution is not the only consideration for in-line SEC measurements. Typically the protein concentration at the point of measurement needs to be sufficiently high to ensure data quality. It is important to observe that the scattering intensity gain outruns the degradation of chromatographic resolution as the load volume is increased. Therefore, for samples that are not expected to scatter well (small molecular size or low concentration), it may be worthwhile to sacrifice resolution for a bigger gain in data quality.

## 3. Data collection   

As an NSLS-II standard, all beamline components are accessible through EPICS. Most components in the sample handler, including the syringe pump for liquid dispensing, the washing diaphragm pump, and the valves that control washing and drying of the flow channels, are connected to a BeagleBone single-board computer that runs *Debian Linux*. We have developed an EPICS IOC using *PCASpy* (Wang, 2018 ▸) to provide EPICS channel access to these components. The HPLC system is controlled by Shimadzu’s proprietary software. The IOC communicates to the HPLC system using the BeagleBone’s GPIO pins that are connected to the contact closure interface on the HPLC system. The Staubli robot is also under EPICS control through an IOC developed by EMBL. With all components under EPICS control, we developed our control code in Python using the *Bluesky* and *Ophyd* libraries developed at NSLS-II (Arkilic *et al.*, 2017 ▸) to take advantage of capabilities such as logging and databroker access.

High-throughput data collection on a PCR tube holder is executed as follows. For each sample in the measurement sequence, the software first identifies the flow channel that the sample will be loaded into. For the purpose of illustration, let us assume it is channel A. The corresponding flow channel A is washed and dried if the dirty flag is true (this is typically not necessary since it should have already been done upon completion of measurement on a previous sample). The sample is then loaded into the entrance of flow channel A, and the dirty flag for channel A is set. Using a separate thread, a background process is started to wash and dry the other flow channel, B, resetting its dirty flag on completion. At the same time, the sample is pushed through flow channel A for X-ray exposure and data collection. Once the wash/dry process for flow channel B is completed, the sample in flow channel A is returned to the sample holder. Data recording on the detectors is aided by the live camera image of the sample as it is pushed through the flow channel. Due to the geometry of the flow channel, the front of the sample flow is not perpendicular to the flow direction, therefore the camera image does not show a clearly defined meniscus. However, the liquid–air interface does produce significant scattering of visible light (see supporting information). This causes a pulse in the values in the standard statistics plugin for the EPICS areaDetector driver, which we use to trigger data recording.

We currently use spreadsheets to schedule automated measurements. A high-throughput run requires a spreadsheet that defines sample holders and sample holder configurations in the storage box. The holder definition specifies the identity (sample name with any other optional information such as sample concentration or description) of the samples in each PCR tube in the holder. If the tube contains a protein solution, the matching buffer is also specified by its name. Before execution of the scheduled measurements, the spreadsheet is validated by software to avoid duplicate sample names and to ensure that there is at least one buffer measurement for each protein solution and that the sample and matching buffer will be measured in the sample flow channel to avoid possible difference in scattering background between channels. On a separate tab in the same spreadsheet, the positions of the sample holders in the storage box for an automated run are defined as a configuration. The holders in a given configuration are measured sequentially. When data collection for each holder is completed, all data produced from samples in the holder are packaged into an HDF5 file and processed automatically (see details in the next section).

Data collection for in-line SEC requires coordination between the beamline and the HPLC control software. For each sample, the beamline announces its readiness to the HPLC system on one of its dry contacts. Once the HPLC system detects this, it proceeds to inject the sample, then notifies the beamline on a second dry contact to start data collection. When the HPLC system completes the SEC run, it again notifies the beamline to stop the current data collection and move to the next sample. This process is implemented as a *Bluesky* scan.

In the scheduling spreadsheet for an automated HPLC run, the user specifies the position of the sample in the autosampler, the load volume and the HPLC method (defined by beamline staff in the *Shimadzu* software) that should be used for the sample. Each SEC run (per sample) lasts ∼20 min for a 3 ml column at a flow rate of 0.35 ml min^−1^ and ∼90 min for a 24 ml column at a flow rate of 0.5 ml min^−1^. The run time corresponds to ∼2× the column bed volume, to minimize interference between consecutive sample loading when a single column is used. With the typical 1 s exposure time, this results in a large number of scattering patterns from each Pilatus detector. The HDF5 packaging of data is therefore performed for each sample, with the UV/IR data attached to the HDF5 file. The scattering data is then processed automatically (azimuthal averaging and merging of data from all detectors) and ready for the user to examine the chromatograms and perform further processing, as discussed in the next section.

## 4. Data processing   

We have expanded the python software initially developed at NSLS (Yang, 2013 ▸) to perform data processing. The new Python package *py4xs* is available on Github as well as Python Package Index (PyPI). For solution scattering, *py4xs* provides capabilities for azimuthal averaging, merging (data from individual detectors) and organizing/accessing data in HDF5 files.

The scattering data are packaged into the HDF5 format using the *suitcase* package developed by NSLS-II. This process involves reading data and metadata (*e.g.* time stamps, states of the beamline components) relevant to the scan back from the data broker, and organizing them into datasets in the HDF5 file. The *py4xs* package adds additional capabilities to store the detector configuration and metadata such as the sample and buffer correspondence, which is initially defined in the scheduling spreadsheet. We also include the HDF5 files and the exported ASCII file information required for publication (Trewhella *et al.*, 2017 ▸) or data deposition (Valentini *et al.*, 2015 ▸), such as the X-ray wavelength and the sample-to-detector distance for each detector.

Azimuthal averaging of the scattering intensity has been optimized to improve processing speed. To reduce the amount of calculation needed for the azimuthal average, the reciprocal coordinate for each detector pixel is calculated first. Azimuthal averaging is then implemented as 2D histogramming of intensity in the pixels based on the *q* values, using the optimized histogram2d() function provided by *numpy* (https://docs.scipy.org/doc/numpy/). Since all detectors are built from the same modules and therefore have the same X-ray sensitivity, when merging the data from different detectors they are scaled strictly based on the sample-to-detector distance, which is derived from scattering data of standard samples (typically silver behenate and barium oxide).

Buffer subtraction is performed automatically based on the height of the water peak at ∼2.0 Å^−1^ (Yang, 2013 ▸). We favor this approach over the use of transmitted beam intensity since it does not require knowledge of the protein-excluded volume (Makowski, 2010 ▸), which needs to be estimated from the protein concentration and specific volume. At LiX, the transmitted beam intensity measured by the intensity monitor built into the beam stop, which is essentially a photodiode watching visible light converted from direct X-ray beam by a YAG crystal, following the design from Australian Synchrotron (Kirby *et al.*, 2013 ▸). Therefore it is an analog signal that can be affected by electronic noise. In comparison, the measurement of the water scattering peak is performed using many (∼10^4^) pixels on the photon-counting WAXS detector. Therefore, the water peak intensity-based approach is also more accurate.

The protein contribution to scattering intensity at ∼2.0 Å^−1^ is orders of magnitudes lower than that typically used in solution scattering data analysis (*q* < 0.3 Å^−1^). But it is always positive, constituting a minute, but finite, contribution to the water peak intensity for protein solution samples. Buffer scattering subtraction by precisely matching the height of the water peaks from the buffer data and the protein solution data would, in effect, over-subtract buffer scattering and result in negative intensity at the water peak position, which is non-physical. Thus, a small manual adjustment of the scaling factor (<0.5%) is allowed to account for the protein scattering contribution, following the criteria that the buffer-subtracted scattering intensity should be positive and smoothly varying, as expected for protein scattering. This adjustment does not alter the data at small scattering angles (*q* < 0.3 Å^−1^), where the protein scattering intensity is much higher, as illustrated in Fig. S3 in the supporting information. A better approach would be to fit the water peak from the buffer data under the protein solution data, which is essentially the goal of adjusting the scaling factor. This is yet to be automated. In practice, we have been using over-subtraction of buffer scattering as an indicator of errors in the measurement. An unusual (>0.5%) scaling factor adjustment or over-subtraction of buffer scattering typically suggests either sample delivery errors (bubbles or insufficient sample in the PCR tube, therefore lower water scattering intensity) during the measurements or mismatched sample and buffer.

**Figure 4:**
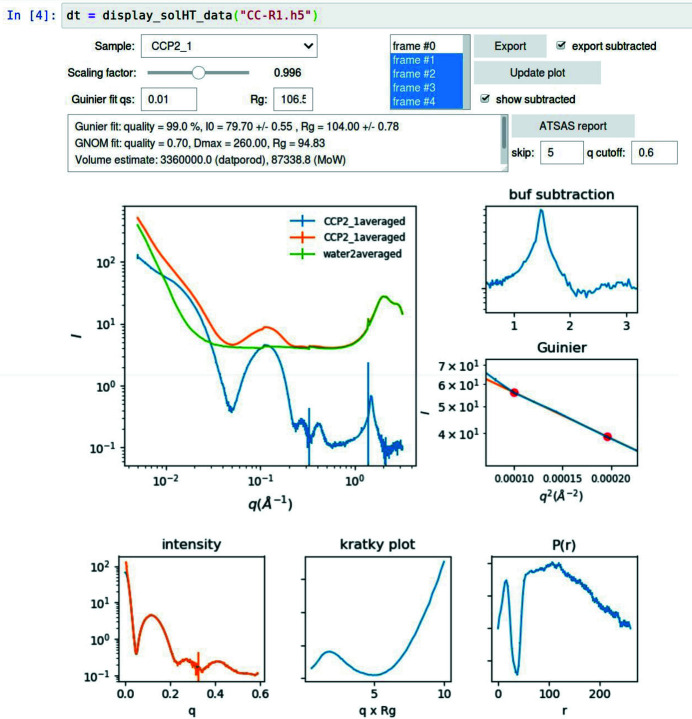
GUI for static solution scattering measurements. The fields in the top portion of the GUI are used for scaling factor adjustment and exclusion of outlier data (the first of the five data frames, or X-ray exposures, was excluded). The middle plots are used to verify the result of buffer subtraction, under the water peak and in the Guinier region. The analysis outputs from *ATSAS* are displayed in the text area and in the bottom plots. The example data shown here are collected from a lipid vesicle sample. The lipid chain–chain correlation peak near 1.5 Å^−1^ is clearly visible. This GUI is provided as part of the *py4xs.notebooks* module.

The processed data are saved back to the same HDF5 file. As an option, the user can choose to export the buffer-subtracted scattering profile as a text file at the time of automated data processing, which can then be further analyzed, for instance, by the *ATSAS* pipeline (Franke *et al.*, 2017 ▸), which is configured to watch for newly exported data. We provide users with a graphical user interface (GUI) as a *Jupyter* notebook (Fig. 4 ▸) to examine the processed data and tweak the scaling factor for buffer subtraction. After buffer subtraction is confirmed, the GUI generates a brief report of the data and calculates the distance distribution function, *P*(*r*), both using the *ATSAS* tools.

**Figure 5:**
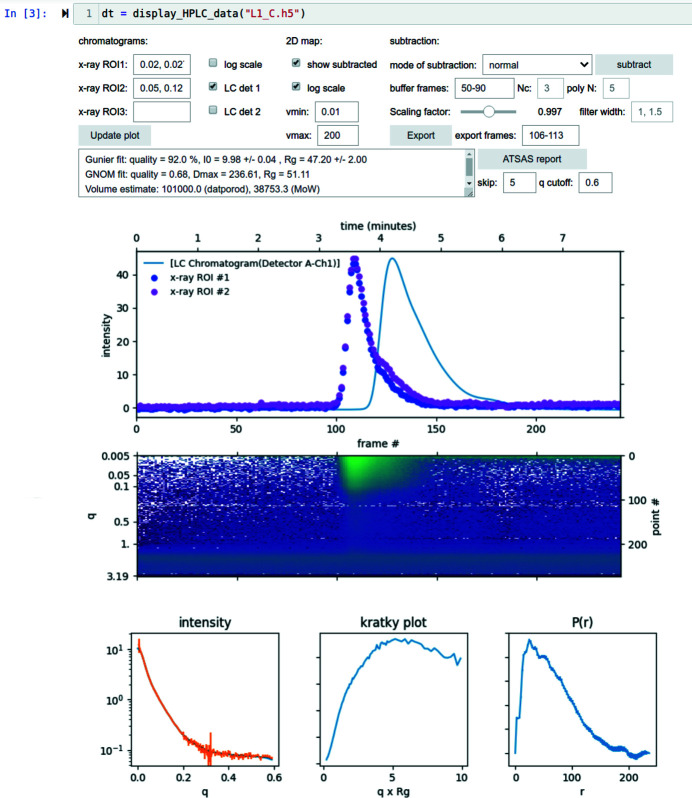
GUI for in-line SEC measurements, provided as part of the *py4xs.notebooks* module. The scattering data are presented at chromatograms of intensity integrated within user-specified *q*-ranges (upper plot, integrated in the ranges 0.02–0.027 Å^−1^ and 0.05–0.12 Å^−1^), as well as a 2D intensity map (lower plot). The offset in time between the X-ray data and the UV detector data (solid line) is due to the different flow rates on the two branches of split sample flows. Buffer scattering subtraction can be performed in either normal mode, where the given buffer frames are averaged to give buffer scattering, or in SVD mode, where the buffer scattering is interpolated as described in the text.

The *Jupyter* notebook GUI for in-line SEC data (Fig. 5 ▸) plots UV/IR chromatograms together with the X-ray intensity integrated within *q*-ranges specified by the user. In addition to these intensity-based 1D chromatograms, we also present the data as a 2D intensity map with coordinates of scattering vector and time. This 2D intensity map, or equivalently the scattering-intensity-based chromatograms at appropriate *q*-values, are informative in distinguishing structural species that exhibit different *q*-dependences in scattering intensity. This is apparent in the example data show in (Fig. 5 ▸).

In principle, a range of data frames can then be chosen as the background for buffer scattering subtraction. However, the scattering background during the SEC run can be time-dependent. An increasing background can be attributed to accumulation of materials on the sample cell (*e.g.* Brookes *et al.*, 2016 ▸). We sometimes also observe decreasing background, possibly due to residual materials from a previous run still coming off the column if there is not sufficient time allowed between samples. Either way, elimination of this drifting background is essential before the component concentration profile can be extracted from the data, either by decomposing the elution curves into peaks of known shapes (Brookes *et al.*, 2016 ▸) or by applying evolving factor analysis (Meisburger *et al.*, 2016 ▸; Hopkins *et al.*, 2017 ▸). In the GUI, we offer an option to use singular value decomposition (SVD) to remove the time-dependent background. To do so, the user specifies a range of data frames within which the sample scattering is expected. SVD is performed on the rest of the data frames. The eigenvalues corresponding to the first few significant eigenvectors are then interpolated within the excluded range of sample frames to give the estimated background scattering.

The data presented in Figs. 4 ▸ and 5 ▸ are selected from our user data and intended to demonstrate the features in the data processing software. For the purpose of providing a reference for data quality comparison, we have included data collected from lysozyme samples in the supporting information.

## 5. Discussion   

The instrumentation and software described here are the products of several iterations of revision. The sample handler itself has operated very reliably in recent experiments for typical protein solutions. Occasionally we still run into minor issues, such as the robot becoming stuck due to overly cautious monitoring of the force-torque sensor, or measurements unable to start as a result of software error in interpreting the scheduling spreadsheet. These issues do not result in sample losses and they are resolved in the process of continual instrument commissioning. For more viscous samples, we have developed a separate setup using non-flowing sample holders that each can hold several samples, also based on mica windows. The samples are measured by scanning to spread out the radiation dose.

We plan to make use of automated data collection and processing to support mail-in access. Given the non-standard format of the PCR tube holder, we have acquired a liquid-handling robot (OT2, Opentron, NY, USA) to transfer samples from standard formats (*e.g.* 96-well) to our sample holders. We also plan to use this robot for generating dilution series and mixing with other chemicals, such as those in compound libraries, to further enable high-throughput measurements. Each PCR tube holder is fitted with a 3D-printed cover to prevent ejection of the PCR tube from the holder during sample handling and provide surfaces to apply a sealing film to minimize evaporation, especially during overnight operations. The cover also has room for a 0.5 inch-square QR code for sample tracking.

An important benchmark of the performance of a solution scattering setup is the sample consumption per static measurement. In principle, this is limited by the radiation dose that the sample can tolerate, which is sample-dependent. In practice, the sample consumption is also limited by the reliability of sample delivery and coordination with the X-ray measurement. The internal volume of our flow channel is ∼10 µl. We have tested different sample load volumes and found that we can successfully collect data with a load volume as low as 20 µl, while still collecting five 1 s exposures per sample. However, radiation damage may become a concern as the total sample volume is reduced, limited by the finite dose tolerance of the sample itself. This is further complicated by the design of the flow cell, in which the sample flow and consequently the sample exposure to X-rays are not uniform. We typically recommend a 50 µl load volume to avoid sample damage. Some of our users prefer to load larger volumes of samples to reduce radiation damage to the samples. The maximum load volume is limited by the tubing internal volume between the end of the injection needle and the entrance of the flow channel, which is 120 µl in our current setup.

We take other measures to limit radiation damage to the samples. As discussed earlier, we intentionally keep the X-ray spot size on the sample fairly large (0.5 mm × 0.5 mm). Since we flow the sample through the beam in all measurements, the benefit of decreased total dose comes from the large vertical beam size (perpendicular to the flow direction), which also results in a large fraction of the sample (∼50%) exposed to the X-rays. Others have taken a different approach to maximize sample utilization by using a small-diameter capillary to shrink the sample size closer to the beam size (Schroer *et al.*, 2018 ▸). We also typically use a relatively high X-ray energy of 13.5 keV to limit X-ray absorption. With an X-ray flux of 2.3 × 10^12^ photons s^−1^ (250 mA ring current), the resulted average dose during a 1 s exposure is 3.5 kGy if the sample is not flowing, but only 0.26 kGy (assuming uniform absorption by water) at the typical flow rate of 10 µl s^−1^ during static measurements. This is reasonable compared with values reported in the literature (Kuwamoto *et al.*, 2004 ▸; Jeffries *et al.*, 2015 ▸; Hopkins & Thorne, 2016 ▸). However, as the NSLS-II ring current is gradually increased towards the design goal of 500 mA, we will need to adjust our data collection strategy to keep the dose within reasonable limit.

Another consideration in static measurements is the throughput of the measurements. As discussed earlier, the bottleneck is the speed of which the samples can be transported to various locations. Some pumps and valves in our current setup are located in a controller separate from the sample handler. The tubing between the two needs to be first filled with water in most operations to ensure responsive sample aspiration and dispensing, but emptied when drying the flow channel. We are planning to relocate some components into the sample handler itself to reduce the measurement time to under 1 min per sample.

Accumulation of damaged sample on the flow cell results in elevated scattering background. Different beamlines have implemented various measures to deal with this issue, from vigorous cleaning (Nielsen *et al.*, 2012 ▸) to utilizing the co-flow device (Kirby *et al.*, 2016 ▸) to minimize the contact of the sample with the capillary cell. We find washing with water adequate during static measurements. This may be attributed to multiple factors: that we use mica windows instead of glass capillaries, the relatively large beam size and the high flow rate of water flow for washing. We measure empty cell and water periodically to keep track of background scattering. In the event that the background scattering becomes unacceptable, a backup flow cell can be swapped in with minimal down time. The radiation dose deposited into the sample is higher in in-line SEC measurements, due to the slower flow rate. Contamination of the window therefore can be more of an issue and is another factor to be considered when planning the experiment.

## 6. Summary   

We have described the instrumentation and the data collection and processing software at the LiX beamline for solution scattering. Further improvements are already being planned. As discussed earlier, we are commissioning a liquid-handling robot to support mail-in measurements, which we expect to start testing with users in 2020. We also plan to add gradient elution capability to the HPLC system, to support ion-exchange chromatography, as reported in the literature (Hutin *et al.*, 2016 ▸), and to explore the structural changes in proteins under different chemical conditions. To keep our users informed of the beamline status and plans, we post user guides, the operating schedule and technical information on our website: https://sites.google.com/view/lixbeamline/. We hold a hands-on training class, which we call the ‘solution scattering workbench’, once every beam cycle and it is open to all users. The training materials are also posted on the website.

## Supplementary Material

Supporting movie. DOI: 10.1107/S1600577520002362/ig5088sup1.mp4

Figures S1 to S5. DOI: 10.1107/S1600577520002362/ig5088sup2.pdf
