# Supplementary material for: Solution scattering at the Life Science X-ray Scattering (LiX) beamline
Source: J Synchrotron Radiat. 2020 Mar 31;27(Pt 3):804–12. doi: 10.1107/S1600577520002362 (PMC7206542; doi:10.1107/S1600577520002362)

JOURNAL OF SYNCHROTRON RADIATION

Volume 27 (2020)

Supporting information for article:

Solution scattering at the Life Science X-ray Scattering (LiX) beamline

Lin Yang, Stephen Antonelli, Shirish Chodankar, James Byrnes, Edwin Lazo and Kun Qian

1. Coverage in reciprocal space by the detectors at LiX

Fig.S1 The scattering data below are collected from a silver behenate standard and show the reciprocal space coverage of the detectors at the LiX beamline. The data have been translated to reciprocal coordinates q_x and q_y (projection of the scattering vector along horizontal and vertical directions in the plane perpendicular to the incident beam). Originally we used two Pilatus 300K detectors for WAXS (left). A new WAXS detector has been installed recently to provide better azimuthal angle coverage at high-*q* (right). This detector, a Pilatus 900K, is customized from an in-vacuum Pilatus 1M, with one module removed. It is sufficient to provide high-*q* data in all measurements and is the only WAXS detector we now use in recent experiments.

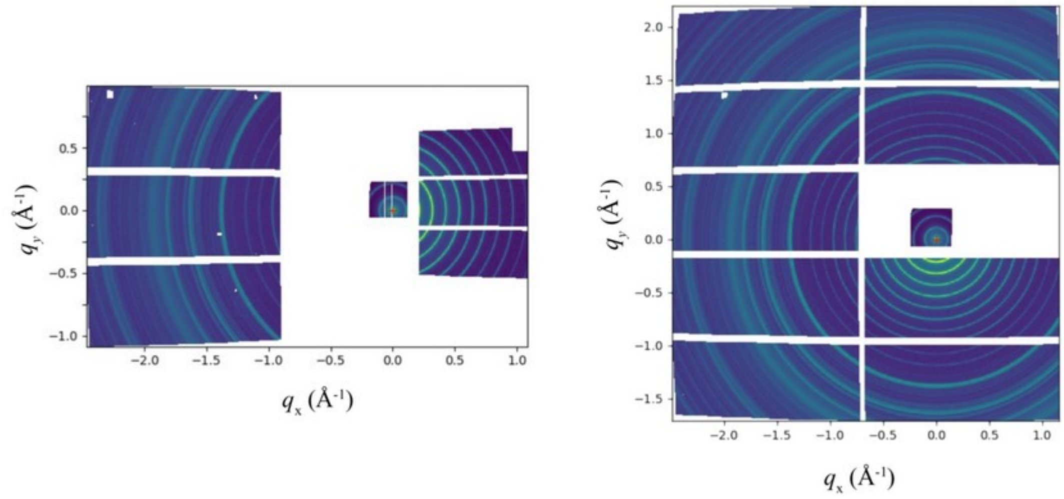

2. Static solution scattering data from Lysozyme

Fig.S2 Scattering data from 5mg/ml lysozyme solution is shown below as a demonstration of typical data quality. The sample was prepared by simply disolvng lysozyme powder (from Chicken egg white, Sigma L6876) in 20mM Tris pH7.5 150mM NaCl buffer without further purification. The plots are generated using the ipython notebook GUI described in the text. The data before buffer scattering subtraction (orange and green curves) show two slight steps at q ~ 0.15 and 0.35 Å^{-1}. They are due to the difference in instrumental scattering background (e.g. air scattering) observed by the SAXS and WAXS detectors. These steps disappear after buffer subtraction (blue curve).

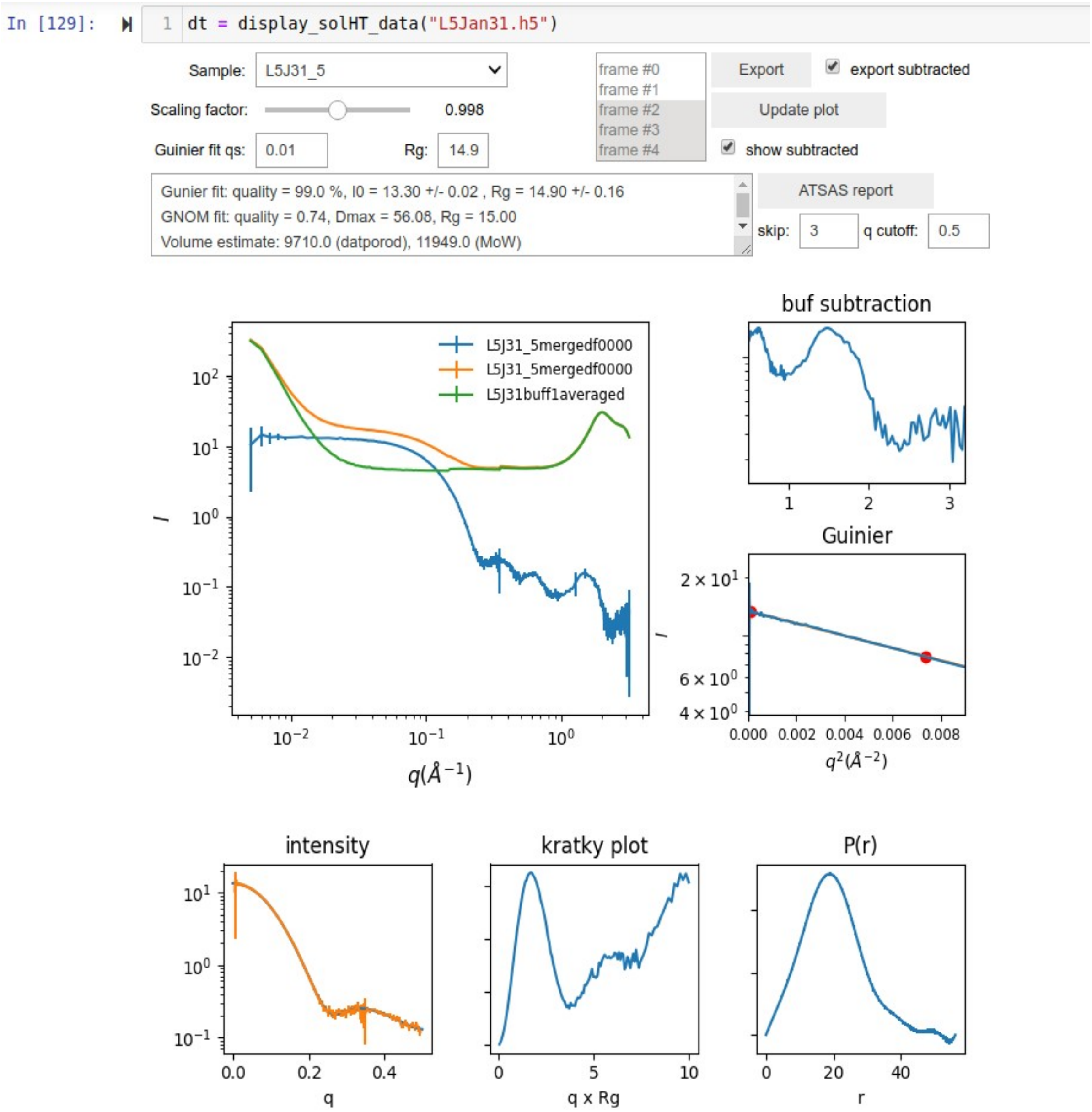

Fig.S3 As discussed in the text, a scaling factor is allowed when subtracting buffer scattering from protein solution scattering. Without this scaling factor (i.e. scaling factor of 1), the subtracted data would include zero or even negative scattering intensity (brown and purple curves in the plot on the left), since scaling of the buffer and protein solution data are based on the intensity integrated within a finite *q*-range at the water scattering peak position. Minor adjustment of this scaling factor improves the high-*q* data without introducing visible changes at low-*q*. This is shown in the Guinier plots (right), in which the black dashed lines is based on the same Guinier fits reported by ATSAS (I_0=13.3 and R_g=14.9 Å). The data have been offset for clarity.

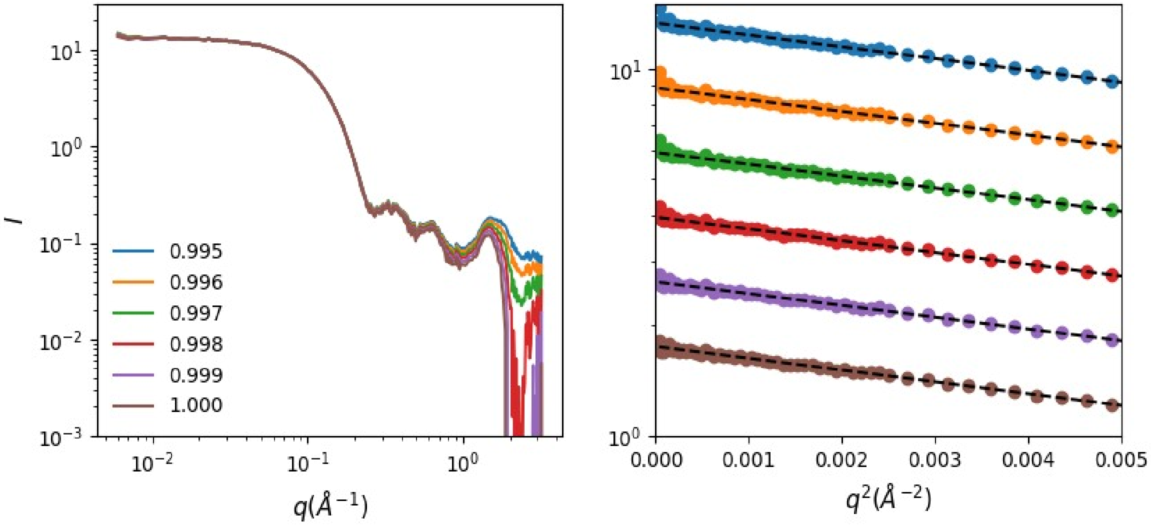

3. In-line SEC data from Lysozyme

Fig.S4 In-line SEC data collected from the same 5mg/ml lysozyme sample used for the static measurement above. The column was a GE Superdex Increase 200 5/150.The sample load volume was 55 µL and the flow rate was 0.5 ml/minute. The plots are generated using the ipython notebook GUI described in the text. There was a slight rise of scattering background during the SEC run (see Fig.S5). The SVD method (described in section 4 in the text) was therefore used for background subtraction. The shape of the peak in the elution curve is asymmetric. But the peak shape on the x-ray and UV detection channels are consistent. The non-ideal shape therefore could be simply due to the quality of the column, which has been in active use at the beamline for a year.

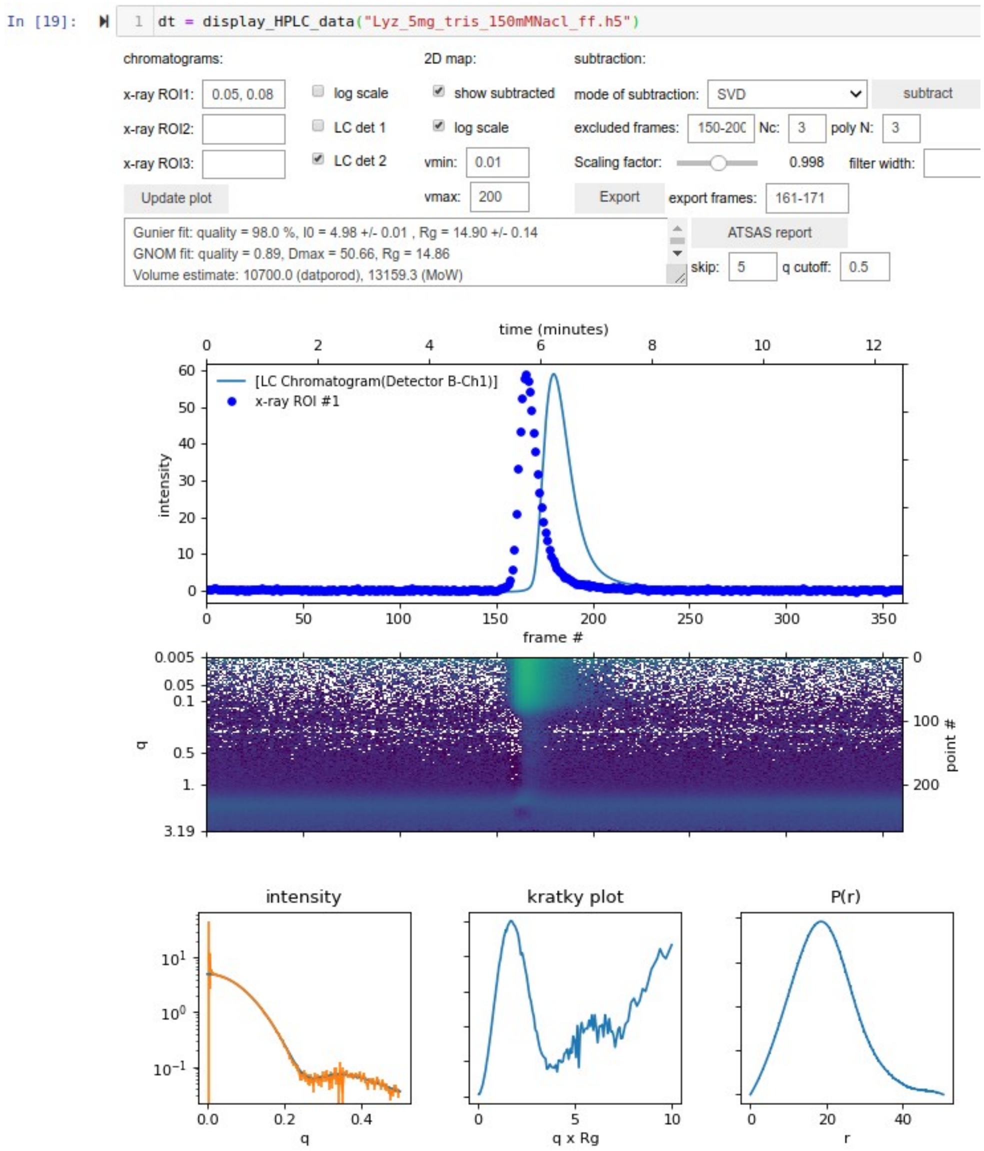

Fig.S5 The same data as shown in Fig.S4, except that background subtraction was performed simply by selecting the data within frames 100-150 as buffer scattering. The 2D intensity map shows the residual scattering intensity at low *q* after the protein peak. However the quality of the buffer-subtracted protein scattering data did not seem to be affected, judging from the I_0 and R_g values reported by ATSAS.

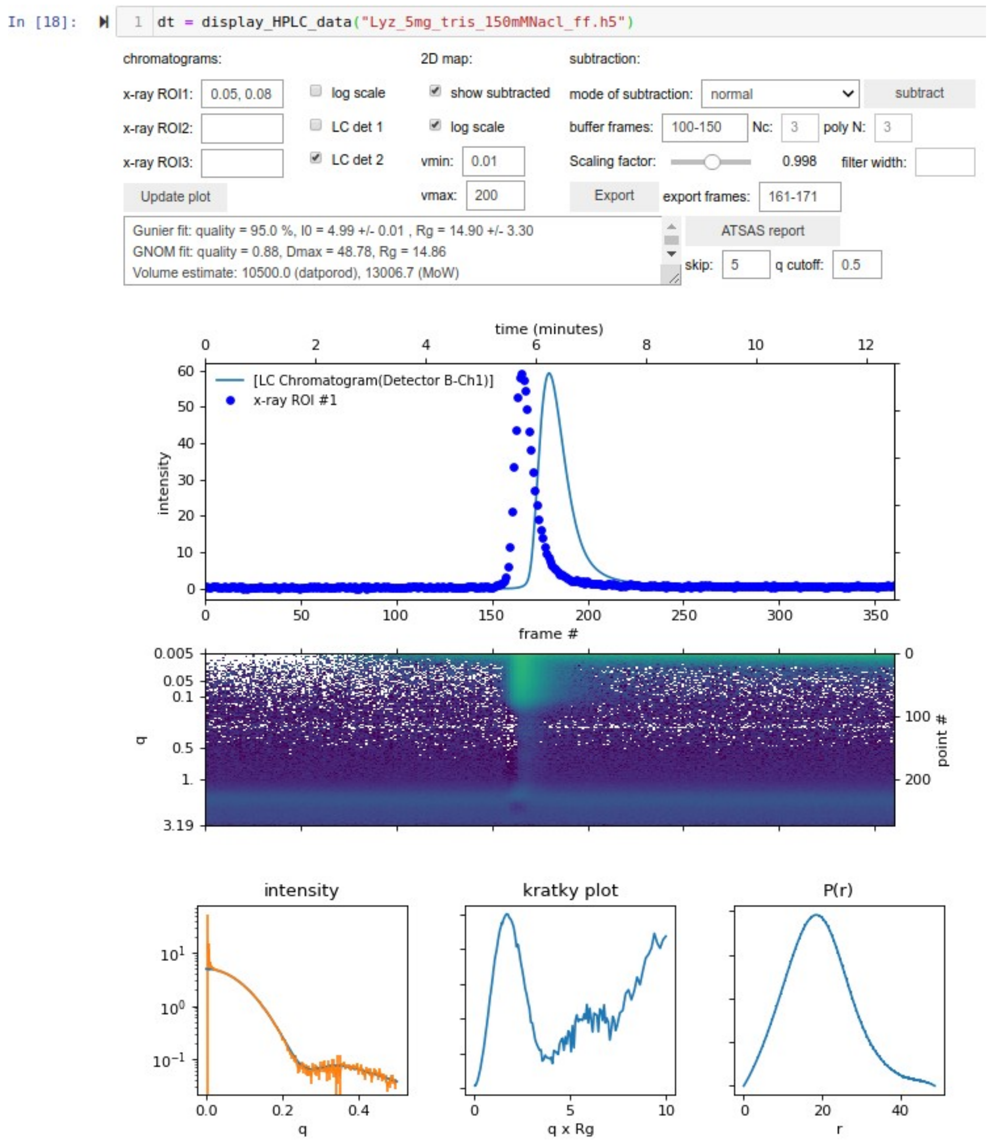

Supplement: Supplementary file 2 [file s-27-00804-sup2.pdf]
